# Cognitive underpinnings of multidimensional Japanese literacy and its impact on higher-level language skills

**DOI:** 10.1038/s41598-021-81909-x

**Published:** 2021-01-26

**Authors:** Sadao Otsuka, Toshiya Murai

**Affiliations:** Department of Psychiatry, Graduate School of Medicine, Kyoto University, 54 Shogoin-kawahara-cho, Sakyo-ku, Kyoto, 606-8507 Japan

**Keywords:** Psychology, Human behaviour

## Abstract

This study aimed to identify the cognitive underpinnings of Japanese kanji abilities and clarify the contributions of kanji acquisition to the development of higher-level language skills based on a three-dimensional view of kanji abilities encompassing reading accuracy, writing accuracy, and semantic comprehension. First, a series of regression analyses was used to identify the multifactorial models of each dimension of Japanese kanji acquisition. These models suggest that, among basic cognitive skills, naming speed, visuospatial processing, and syntactic processing underpin kanji abilities in a dimension-specific manner, whereas phonological processing is a common factor. Second, although all the dimensions of kanji abilities predicted acquired verbal knowledge equally, writing skills on the text level, measured as idea density, were only predicted by the writing dimension (indirectly via acquired knowledge). Our findings represent the first evidence of the dimension-specific relationships of the three dimensions of Japanese kanji abilities with their cognitive predictors, as well as with higher-level language skills. They suggest the importance of handwriting acquisition during school years for the development of language skills through to adulthood. Finally, taking the seminal “Nun study,” which suggests that higher idea density is protective against dementia, into account, we propose a theoretical framework for the lifelong trajectory of literacy acquisition.

## Introduction

### Japanese literacy and kanji abilities

The Japanese writing system is composed of two types of script, i.e., kana and kanji. The former is phonographic, in which a letter is mapped onto a unit of sound, as in English. The latter is logographic, in which a character is mapped onto a unit of meaning, as in Chinese. Like alphabetic orthographies with 24–36 letters^1^, the former consists of only 71 letters, which are visually simple, and all kana letters are acquired by the first year of elementary school. In contrast, the latter contains more than 5,000 characters, which vary in visual complexity, as is the case with Chinese orthography^2^. Kanji characters are acquired gradually based on school grade throughout Japan, in strict accordance with the school curriculum guidelines developed by the Ministry of Education, Culture, Sports, Science and Technology^3,4^. In the first grade, children learn 80 characters (from a total of 2,136 daily-use characters). They are expected to master 1,006 characters by the end of the sixth grade, and to be able to read all daily-use kanji, including visually very complex characters such as the kanji 鬱 (*u-tsu*, depression), and to be able to write most of them by hand before high school graduation^3,4^. In Japanese documents, most nouns, and the roots of most verbs, adjectives, and adverbs are generally written with kanji characters, whereas kana letters are used adjunctively for function words and inflectional endings. Although the Japanese language can be written exclusively with kana letters, many homophones in the Japanese language are represented with the same kana letters; therefore, texts written with only these letters are generally much less readable. Japanese kanji characters make homophones discriminable, and mastery of a larger number of kanji characters and words guarantees that one will be able to read and write more complex sentences with a higher proportion of abstract words.

Literacy acquisition in any language is underpinned by basic cognitive skills and can support the development of higher-level language skills. This means that literacy can be a mediator between early cognitive skills and intellectual abilities in adulthood. Japanese literacy is considered to depend largely on skills for managing kanji words, which are acquired gradually over time and are used in preference to kana. However, the cognitive-developmental bases of Japanese kanji abilities are unclear, whereas it has been repeatedly reported that kana acquisition is strongly predicted by phonological processing^5–7^, as is also the case with English^8,9^. The cognitive underpinnings or demands of kanji acquisition that lasts until adulthood are considered to involve more slowly developing functions than, or at least to be partly different from, those of kana acquisition. Given the higher prevalence of problems in literacy acquisition in kanji than in kana among Japanese children^10^, identifying the cognitive underpinnings of Japanese kanji abilities is important for reinforcing therapeutic or educational strategies for these problems. In addition, literacy on the word level is one of the essential bases supporting knowledge acquisition at school or elsewhere, as well as the development of reading/writing skills on the text level. Therefore, from the perspective of the development of language skills, this study investigated the relationship of kanji abilities with their cognitive underpinnings as well as with higher-level language skills in Japanese university students who had completed 12 school years of kanji education.

### Cognitive underpinnings of literacy

First, to identify the cognitive predictors of kanji abilities, it is necessary to consider multiple and interacting risk/protective factors of literacy problems whose etiology is complex and multifactorial, such as developmental dyslexia^11,12^. Cross-cultural investigations have established the importance of phonological processing as one of the key predictors of literacy acquisition in alphabetic orthographies (Dutch, German, English, Finnish, French, Hungarian, and Portuguese)^13,14^, as well as in logographic Chinese^15^. A single-deficit phonological theory of literacy problems was formerly predominant; however, emerging evidence suggests the possible contribution of deficits in visuospatial processing, including visuomotor processing speed, to literacy problems in alphabetic^8,9,16–19^ and Chinese orthographies^20^. Additionally, it has long been hypothesized that naming speed is a predictor of reading acquisition^8,9,21^, and it has also been reported to be related to spelling ability in alphabetic orthographies^13^. These cognitive domains have been shown to contribute differently to reading and writing^13,23^.

With reference to Japanese kanji abilities, a few studies replicated results showing significant relationships between the three cognitive domains mentioned above and early acquisition of Japanese kanji reading/writing accuracy, with a relatively strong contribution of visuospatial processing^6,10,22^. However, to the best of our knowledge, no study has yet investigated the cognitive underpinnings of the semantic dimension of Japanese literacy skills, presumably because of the young age of the participants in the previous studies. The three-dimensional view of kanji abilities, in which semantic comprehension interacts with but is distinct from accuracy in reading and writing, can be supported by neuropsychological evidence^24–26^, as well as the results of a factor-analytic investigation into a large database of test-takers’ performance details on the Japan Kanji Aptitude Test (*Nihon Kanji Noryoku Kentei*: Kanken)^27^. As suggested by the results of previous studies of alphabetic and Chinese orthographies examining the cognitive underpinnings of the abilities to read and comprehend sentences or a text^28,29^, syntactic processing is considered to be a likely predictor of the semantic dimension of kanji acquisition in Japanese. Thus, we investigated the predictive relationships between basic cognitive skills, including phonological, visuospatial, and syntactic processing, and naming speed, and the dimension of semantic comprehension as well as reading/writing accuracy in Japanese kanji abilities. As might be expected, the writing dimension of literacy was assessed in this study using tasks that require participants to write words accurately by hand rather than to type them, in common with the studies referred to above.

### Impact of literacy on higher-level language skills

Second, regarding the outcomes of later linguistic development, empirical evidence shows that literacy skills can predict the acquisition of verbal knowledge during the school years. For example, the Matthew effect suggests that good readers gain vocabulary, as well as reading skills, more rapidly^30–32^. In addition, it has been reported that scores on the Information subtest of the Wechsler intelligence scales in children/adults with developmental dyslexia are significantly lower than those in typically developing people^33,34^. Furthermore, literacy skills have been reported to be related to idea density, which represents the linguistic complexity of written or spoken output^35^. Idea density has been the subject of widespread attention in the context of its possible function as a protective factor against age-related cognitive decline and Alzheimer’s disease in late life. The seminal “Nun study” retrospectively analyzed autobiographies that had been written by nuns in their young adulthood (mean age of 22 years) and showed that higher idea density predicted intact cognition in later life, regardless of the presence or absence of Alzheimer’s disease lesions^36,37^. This relationship between higher idea density in young adulthood and intact cognition in later life was replicated in a study that analyzed medical school admission essays (mean age of 22 years)^38^. These findings suggest that attainment of higher language proficiency before and during young adulthood may protect against cognitive decline and dementia in late life, and in addition, seem to validate the notion that idea density can be a measure of highly sophisticated skills. From a developmental perspective, higher-level language skills such as idea density are considered to be partly underpinned by basic literacy skills, or, more specifically, idea density may be encouraged by the accumulation of verbal knowledge, which is based on literacy acquisition.

In recent decades, the rapid spread of digital writing devices such as computers and smartphones^39^ has drastically reduced the frequency of handwriting^40^, resulting in a dimension-specific reduction of kanji writing accuracy in Japanese adults^27^. When using these devices, we first write with kana letters and then convert them into kanji words by choosing one option from many homophones. When doing so, we recognize, not recall, the form of kanji characters. In addition, we can write Japanese words with kana whenever we cannot write kanji characters, which are large in number and visually complex, correctly. Therefore, less frequent handwriting in Japanese can easily lead to less correct handwriting of kanji. Considering these effects of environmental changes, it seems important to examine the impacts of each dimension of those abilities on higher-level language skills.

### Aim of the study

This study aimed to clarify the relationships among Japanese kanji abilities, their cognitive underpinnings, and higher-level language skills from the perspective of the development of language skills. Considering the dimension-specificity of kanji acquisition, this cross-sectional study started by identifying the risk/protective factors that account for the individual differences in each of the three dimensions of kanji abilities, i.e., reading accuracy, writing accuracy, and semantic comprehension, in Japanese university students. Then, we examined the contributions of each dimension of kanji acquisition to higher-level language skills, i.e., acquired knowledge and idea density. The hypotheses tested in this study were that (1) multidimensional kanji abilities can be predicted by multiple cognitive factors; (2) those cognitive skills predict kanji abilities in a dimension-specific manner; (3) the relationships between kanji abilities and acquired knowledge are not correlations but predictions from the former to the latter; (4) kanji abilities mediate the relationships between their cognitive predictors and higher-level language skills; (5) kanji abilities predict idea density dimension-specifically and indirectly via acquired knowledge.

## Methods

### Participants

The present study included 30 university students (28 undergraduates and 2 graduates; mean age ± SD = 19.87 ± 1.25 years; 15 females and 15 males) recruited from several universities in Kyoto and Osaka, Japan. The sample size was calculated a priori using the GPower 3.1.9.2 (Kiel University, Germany) based on an *α* = 0.05, power of 80%, and a large effect size (*f*^2^ = 0.35) for identifying two predictors using a multiple linear regression analysis. The full-scale, verbal, and performance intelligence quotients (IQ) of all participants were measured using the Japanese version of the Wechsler Adult Intelligence Scale, third edition (WAIS-III)^41,42^. The IQs of all participants were normal or higher (IQs ≥ 72), and the variance and width of the distribution of the scores were substantially large (SDs ≥ 11.95; width of the IQ ranges ≥ 52), as expected. Moreover, to estimate the literacy achievement level of the participants, we used the reading and handwriting subtests of the Japanese version of the Kaufman Assessment Battery for Children, second edition (KABC-II)^43^. The KABC-II is standardized for children aged 2–18 years. Thus, we applied scaled scores for 18-year-olds to all participants, even though this may have meant slightly overestimating the achievement level of the participants. The scores for reading and handwriting were normal or higher (scaled scores ≥ 8), and the variance and width of the distribution of the scores were substantially large (SDs ≥ 2.11; width of the scaled score ranges ≥ 7). The demographic characteristics and the scores on WAIS-III and KABC-II of the participants are provided in Table 1.

**Table 1:** Demographic characteristics, intelligence, and literacy achievement level of participants.

	Mean	SD	Range
Sex (% male)	50%		
Age (years)	19.87	1.25	18–23
Education (years)	13.07	1.23	12–16
**Intelligence (WAIS-III)**			
Full-scale IQ	116.13	11.95	82–134
Verbal IQ	118.77	13.81	91–144
Performance IQ	109.43	12.15	72–134
**Literacy achievement level**			
KABC-II Reading (scaled score)	12.77	2.11	8–15
KABC-II Handwriting (scaled score)	13.23	2.45	8–17

Note: *n* = 30.

*SD* standard deviation, *WAIS-III* Wechsler Adult Intelligence Scale, third edition, *IQ* intelligence quotient, *KABC-II* Kaufman Assessment Battery for Children, second edition, the mean IQ ± SD of the WAIS-III is 100 ± 15, the mean scaled score ± SD of KABC-II is 10 ± 3.

All procedures in this study were approved by the Ethics Committee of Kyoto University Graduate School and Faculty of Medicine and were performed in accordance with the ethical standards in the 1964 Declaration of Helsinki and its later amendments. All participants provided written informed consent to participate in the study.

### Measures

All cognitive tasks and neuropsychological tests were administered individually by a clinical psychologist trained in standardized testing procedures. The written exams, including a kanji exam and an essay task for measurement of idea density, were also conducted individually and overseen by a clinical psychologist or one of two undergraduate research assistants to prevent examinees from using their smartphones during the exams. Considering that fatigue could influence task performance, the measures described below were divided into two parts and implemented over two consecutive or non-consecutive days within 21 days, with the exception of one individual who participated with an interval of 51 days between the two parts of the test.

#### Kanji abilities

To measure kanji abilities, we used the 2016 past examination papers for the Kanken, whose three-dimensional structure, including reading accuracy, writing accuracy, and semantic comprehension, was established in our factor analytical study^27^. The Kanken was first offered in 1975 and is the most popular kanji exam in Japan, with approximately 2 million people taking it voluntarily or semi-voluntarily in 2016. From the 12 levels of difficulty provided by Kanken, ranging from the easiest (Level 10) to the most difficult (Level 1, including Pre-2 and Pre-1), we employed the Level Pre-2 exam paper (mastery of 1951 daily-use kanji: high school level). Additionally, considering the possible ceiling effects in the Reading subtest^27^, we also conducted that subtest from the Level 2 exam paper (mastery of all 2136 daily-use kanji; high school graduation level). The results showed that only one participant achieved a perfect score on the Level 2 Reading subtest, whereas six participants did so on that of Level Pre-2. Thus, we eventually analyzed the scores on the Level 2 Reading subtest, in addition to the other subtests of the Level Pre-2 exam papers. All participants completed those paper-and-pencil exams within 60 min (mean ± SD = 32.50 ± 7.09 min; the range was from 19 to 46 min), which was the time limit for the official Kanken exam.

##### Reading accuracy

The dimension of reading accuracy was assessed using the Reading subtest. This subtest requires examinees to write the correct pronunciation of a marked kanji word (i.e., convert it to kana) appearing in each of 30 sentences, taking context into consideration. As previously discussed, a kanji word can be written alternatively with kana letters, which have highly regular letter–sound correspondences. Thus, this conversion from kanji characters to kana letters is usually used in kanji education in Japan. Each correct item was given a score of 1, adding up to a maximum of 30 (0–30)^27^.

##### Writing accuracy

The dimension of writing accuracy was assessed using the following five subtests. We used the sum of the scores on these subtests as the score for this dimension (0–110). The Antonyms/Synonyms subtest required the participants to choose an antonym or synonym for each of ten two-character kanji compounds from kana words and write it correctly in kanji. Ten options of kana words were prepared. Each correct item was given a score of 2, adding up to a maximum of 20. In the Homophones subtest, the participants were required to differentially write two homophones of kanji words that were written as underlined kana letters in each of five pairs of sentences. Each correct item was given a score of 2, adding up to a maximum of 20. The Error Correction subtest required the participants to identify a homophonic error in a kanji character in each of five sentences and write the correct one. Each correct item was given a score of 2, adding up to a maximum of 10. In the Kana Suffixes subtest, the participants were required to write a correct kanji character and a kana suffix accompanying it based on marked kana letters in each of five sentences. Each correct item was given a score of 2, adding up to a maximum of 10. The Writing subtest required the participants to write a correct kanji word that was written as marked kana letters in each of 25 sentences. Each correct item was given a score of 2, adding up to a maximum of 50^27^.

##### Semantic comprehension

The dimension of semantic comprehension was assessed using the following four subtests. We used the sum of the scores on these subtests as the score for this dimension (0–60). The Radicals subtest required the participants to extract a radical from each of ten kanji characters. Radicals are the visual components of kanji characters, most of which represent the semantic category, e.g., the left part of the kanji 海 (*umi* or *kai*, sea) is regarded as the radical 氵 (*sanzui*), meaning “water” or “fluid.” In general dictionaries of Japanese kanji, 214 radicals are used for classifying kanji characters and each kanji is assigned one radical. Each correct item was given a score of 1, adding up to a maximum of 10. In the Compounds Structure subtest, the participants were required to classify ten two-character kanji compounds into five categories based on their structure. The categories included cases where the two characters have a similar meaning, the two characters have the opposite meaning, the former modify the latter, the latter is an object/complement of the former, and the former deny the meaning of the latter. Each correct item was given a score of 2, adding up to a maximum of 20. The Compounds Completion subtest required the participants to complete ten four-character kanji compounds by choosing one that precedes or follows each of ten two-character kanji compounds from kana words and converting kana to kanji. Ten options of kana words were prepared. Each correct item was given a score of 2, adding up to a maximum of 20. In the Compounds Meaning subtest, the participants were required to choose one option that represented the meaning of each of five sentences from ten four-character kanji compounds in subtest 4. Each correct item was given a score of 2, adding up to a maximum of 10^27^.

#### Cognitive skills

Drawing on the findings of previous studies^6,8–10,13–23,28,29^, we selected and measured cognitive skills in the domains of phonological processing, naming speed, visuospatial processing, and syntactic processing. To assess basic cognitive skills that are substantially independent of each other and likely underpin literacy acquisition, we employed simple tasks whose targets were clear.

##### Phonological processing

We used the Checking Sounds subtest of the Comprehensive Assessment of Reading Domains (CARD)^44^ to measure phonological awareness. In this task, the participants listened to two voices that read out each of 30 words (3–5 morae) in sequence, with one of the morae not read by the second voice; they were then asked to identify the location of the mora that was canceled. In this test, which was standardized for elementary school children, at normal speed, the audio stimuli that sequentially present words are played over about 215 s. However, in the present study, we played the stimuli at double speed to make the task more challenging for university students based on the results of our preliminary experiments. Taking the number of correct answers as a measure of accuracy, the high level of accuracy (mean = 97.23%) of the participants’ performance on this subtest appeared to support the assumption that this modification would not diminish the capability of this test.

To measure sound–letter decoding, we used the Listening subtest of the CARD^44^. In this task, the participants were asked to listen to a voice that read out each of 30 non-words (3–5 morae) and to choose one non-word that represented the sound heard from five options, each of which was written with 3–5 kana letters. In this test, which was standardized for elementary school children, the audio stimuli that sequentially present non-words are played over about 155 s at normal speed. However, to make the task more challenging for university students, we played the stimuli at double speed in the present study based on the results of our preliminary experiments. Taking the number of correct answers as a measure of accuracy, the high level of accuracy (mean = 92.43%) of the participants’ performance on this subtest appeared to support the assumption that the modification would not diminish the capability of this test.

To measure phonological short-term memory, the forward condition of the Digit Span subtest of the WAIS-III^41,42^ was used. In this task, participants were required to repeat a sequence of digits presented orally by the examiner. The measure used was the raw score.

##### Naming speed

We used the Rapid Automatized Naming tests^45^ to measure naming speed. In this task, the participants were asked to name a random sequence of symbols (i.e., objects, colors, numbers, and alphabetical letters) as quickly and accurately as possible. We administered this task in Japanese and used the sum of the time (in seconds) taken to name all the symbols in the Object, Color, and Number subtests as the measure. Considering the time needed to correct errors spontaneously, we adjusted the score by adding the number of errors overlooked to the time.

To measure letter–sound decoding, the Non-word Reading task^46^ was used. In this task, participants were required to read 30 non-words written with four kana letters (3–4 morae) aloud as quickly and accurately as possible. The time (in seconds) taken to read all the non-words was used as the measure, and we adjusted the score by adding the number of errors overlooked to the time.

##### Visuospatial processing

We used the Kana subtest of the Visual Cancellation task of the Clinical Assessment of Attention^47^ to measure visual attention. This task asked participants to look through a random sequence of kana letters only once and cross out a target letter that appeared repeatedly within the sequence as quickly and accurately as possible. The measure used was the time (in seconds) taken to complete the task. Considering the shorter completion time of the 17 participants who made more than one error (mean ± SD = 96.82 ± 20.38) compared with the 13 participants who made no errors (mean ± SD = 105.54 ± 22.05) and the number of errors in the former (mean ± SD = 2.06 ± 1.38), we adjusted the score by adding ten times the number of errors to the time (consequently, mean ± SD score in the participants who made more than one error = 118.59 ± 26.58).

To measure visual short-term memory, the forward condition of the Visuospatial Span subtest of the Japanese version of the Wechsler Memory Scale, Revised (WMS-R)^48,49^ was used. In this task, after the examiner had tapped the cubes in a predetermined sequence, participants were asked to repeat the sequence. The raw score was used as the measure.

To measure visual long-term memory, we used the Rey Complex Figure Test^50^. This task requires participants to draw a design relying on memory 3 min (immediate recall) and 30 min (delayed recall) after completing a trial copy. The measure used was the raw score of the delayed recall.

To measure visuospatial perception, we used the unsegmented condition of the Block Design task developed by Shah and Frith^51^. In this task, participants were asked to replicate designs using four blocks as quickly as possible, as with the Block Design subtest of the WAIS-III. The total time (in seconds) to construct all six designs was used as the measure.

To measure visuomotor processing speed, the Digit Symbol subtest of the WAIS-III^41,42^ was used. This task requires participants to copy symbols paired with digits as quickly as possible in the empty boxes below a random sequence of digits within 120 s. The measure used was the raw score.

In addition, we used the Purdue Pegboard Test^52^ to measure manual dexterity. This task requires participants to insert small metal rods into a row of holes using their right, left, or both hands. Because all of the participants were righthanded, the number of rods inserted within 30 s using their right hand was used as a measure. In this study, we related the demands of eye—hand coordination in this task, which could have an impact on literacy acquisition, to visuospatial processing.

##### Syntactic processing

The Sentence Reading Part 2 subtest of the CARD^44^ was used to measure syntactic processing. In this task, the participants were asked to read a sentence and then judge whether the five sentences following that sentence were semantically identical with it. Those five sentences differed in their syntactic structures and function words from the sentence read first. The measure used was the time (in seconds) taken to answer all 50 questions (ten sets of five sentences). Considering the time for the participants to complete the task (mean ± SD = 188.00 ± 48.20), we adjusted the score by adding five times the number of errors to the time.

#### Higher-level language skills

##### Acquired knowledge

Following the method suggested by Bannatyne^53^, we employed the sum of the scaled scores on the Vocabulary, Arithmetic, and Information subtests of the WAIS-III as the measure of acquired knowledge. The Vocabulary subtest asked participants to explain orally the meaning of words presented both orally and in written form. This test is similar to the semantic subtests of kanji abilities in requiring semantic knowledge but differs in terms of not requiring literacy skills from those subtests. In fact, the scores on the Vocabulary subtest in dyslexic children are not always lower than those in control children^34,54,55^, suggesting that vocabulary and literacy skills are not identical. In the Arithmetic subtest, participants mentally solved arithmetic questions presented orally. This test assumes not only working memory but also knowledge of the rules of arithmetic. The Information subtest asked for knowledge generally acquired at school.

##### Idea density

We calculated idea density scores in essays written by participants using the CPIDR 5.1.4637.21009 (Computerized Propositional Idea Density Rater, pronounced “spider”) software program^56^, which automatically determines the idea density of an English text based on part-of-speech tags and rules for adjusting the count of propositions, i.e., verbs, adjectives, adverbs, prepositions, and conjunctions (for more details, see the CPIDR 5.1 User Manual)^56^. In short, idea density was measured as the ratio of expressed propositions to the total number of words in an English text^36^. Before calculating idea density, in accordance with a suggestion from a previous study that demonstrated the feasibility of machine translation–based measurement of idea density in Japanese texts^57^, we translated the Japanese text written by participants into English using Google Translate. The participants were asked to write an essay concerning “general activities and interests” in 600–800 characters within 30 min using Microsoft Word on an offline PC. The thesis of an essay was determined according to the method used in a previous study that calculated idea density in essays written by university students^38^. All but four participants completed this task within 30 min, and the time was extended up to 35 min when not completed (mean ± SD = 23.73 ± 5.97 min; the range was from 10 to 35 min).

### Statistical analyses

The data were analyzed in three steps. All statistical analyses were conducted using the R version 3.5.3^58^ software environment. The statistical testing was two-tailed, and α was set at 0.05.

First, to identify the cognitive factors accounting for the individual differences in multidimensional kanji abilities, we performed simple linear regression analyses between all measures of cognitive skills as independent variables (predictors) and each dimension of kanji abilities as the dependent variable (outcome). Then, to identify the best predictive models for each dimension of kanji abilities, multiple linear regression analyses were conducted between significant predictors and kanji abilities.

Second, we assessed goodness of fit for each of the three models of relationships among each dimension of kanji abilities, their cognitive predictors, and acquired knowledge or idea density with path analyses using maximum likelihood estimation. The first model assumed the predictions from kanji abilities to acquired knowledge, whereas the second model assumed the correlations between them. The third model hypothesized that each of the kanji abilities could directly predict idea density. The following indices of model fit were employed: the traditional *χ*^2^ test, the root mean square error of approximation (RMSEA) with a 90% confidence interval, the comparative fit index (CFI), the Tucker-Lewis index (TLI), the standardized root mean square residual (SRMR), and Akaike’s information criteria (AIC). RMSEA values < 0.05 suggest a good fit, and values < 0.08 are considered acceptable. We also calculated *p*-values for the test of the close-fit hypothesis that RMSEA ≤ 0.05. This one-sided null hypothesis should be adopted (i.e., *p* close ≥ 0.05) for a good fit^59^. The CFI and TLI values should be > 0.95, the SRMR values should be < 0.08 for a good fit, and lower AICs indicate relatively better fit^60–62^. In addition, we conducted a bootstrapping method to test the mediation path in the models with a good fit.

Then, for testing the direct and indirect effect (via knowledge acquisition) of kanji abilities on idea density, we performed linear regression analyses with a bootstrapping method. An estimate of the indirect effect was the mean computed using 1000 bootstrap samples.

## Results

### Cognitive predictors of kanji abilities

Means, standard deviations, and range of scores on all measures are shown in Table 2, and the results of simple linear regression analyses between cognitive skills as predictors and three dimensions of kanji abilities as outcomes are presented in Table 3. These analyses showed that better performances on sound–letter decoding (*β* = 0.57, *t*(28) = 3.66, *p* = 0.001), rapid automatized naming (*β* = − 0.46, *t*(28) = − 2.77, *p* = 0.010), letter–sound decoding (*β* = − 0.45, *t*(28) = − 2.68, *p* = 0.012), and syntactic processing (*β* = − 0.54, *t*(28) = − 3.43, *p* = 0.002) significantly predicted higher scores in reading accuracy kanji ability. However, higher scores in writing accuracy were found to be predicted by better performance on phonological awareness (*β* = 0.44, *t*(28) = 2.62, *p* = 0.014), sound–letter decoding (*β* = 0.57, *t*(28) = 3.63, *p* = 0.001), visual short-term memory (*β* = 0.45, *t*(28) = 2.65, *p* = 0.013), visuospatial perception (*β* = − 0.53, *t*(28) = − 3.27, *p* = 0.003), and syntactic processing (*β* = − 0.47, *t*(28) = − 2.80, *p* = 0.009). Higher scores on semantic comprehension were predicted by better performance on phonological short-term memory (*β* = 0.41, *t*(28) = 2.35, *p* = 0.026), visuospatial perception (*β* = − 0.43, *t*(28) = − 2.53, *p* = 0.017), and syntactic processing (*β* = − 0.50, *t*(28) = − 3.07, *p* = 0.005). No other relationship between cognitive skills and kanji abilities was significant (all *p* ≥ 0.06). Additionally, simple linear regression analyses showed that sex, age, and years of education did not predict any dimension of kanji abilities, as well as acquired knowledge and idea density (all *p* ≥ 0.12).

**Table 2:** Scores on language and cognitive measures in participants.

	Mean	SD	Range
**Kanken: Kanji abilities (raw scores)**			
Reading accuracy	24.33	3.74	15–30
Writing accuracy	73.47	24.98	10–102
Semantic comprehension	41.17	7.99	22–53
Total score	138.97	33.94	51–183
**Cognitive skills**			
Phonological processing			
Phonological awareness (raw score)	29.17	1.12	26–30
Sound–letter decoding (raw score)	27.73	2.66	20–30
Phonological short-term memory (raw score)	12.73	2.30	8–16
Naming speed			
Rapid automatized naming (s)	69.93	10.58	53–98
Letter–sound decoding (s)	26.93	6.88	16–44
Visuospatial processing			
Visual attention (s)	112.93	25.18	72–166
Visual short-term memory (raw score)	11.43	1.74	8–14
Visual long-term memory (raw score)	27.92	5.31	15–36
Visuospatial perception (s)	44.57	14.19	29–105
Visuomotor processing speed (raw score)	98.70	15.95	65–129
Manual dexterity (raw score)	13.30	1.78	10–17
Syntactic processing			
Syntactic processing (s)	205.00	71.42	113–461
Acquired Knowledge (WAIS-III)	40.23	7.39	23–51
Vocabulary (scaled score)	13.50	2.65	9–19
Arithmetic (scaled score)	13.83	2.98	7–18
Information (scaled score)	12.90	3.17	4–16
Idea density	0.52	0.03	0.44–0.56

Note: *n* = 30.

*SD* standard deviation, *Kanken* Japan Kanji Aptitude Test.

**Table 3:** Results of simple linear regression analyses including cognitive skills as predictors and kanji abilities as outcomes.

	Reading accuracy			Writing accuracy			Semantic comprehension		
	*Β*	*t*(28)	*p*	*β*	*t*(28)	*p*	*β*	*t*(28)	*P*
**Phonological processing**									
Phonological awareness	0.25	1.37	0.183	0.44	2.62	0.014	0.15	0.81	0.424
Sound–letter decoding	0.57	3.66	0.001	0.57	3.63	0.001	0.23	1.25	0.223
Phonological short-term memory	0.23	1.25	0.220	0.30	1.69	0.102	0.41	2.35	0.026
**Naming speed**									
Rapid automatized naming	− 0.46	− 2.77	0.010	− 0.20	− 1.05	0.302	− 0.16	− 0.87	0.392
Letter–sound decoding	− 0.45	− 2.68	0.012	− 0.21	− 1.13	0.269	− 0.07	− 0.36	0.720
**Visuospatial processing**									
Visual attention	− 0.31	− 1.72	0.096	− 0.34	− 1.90	0.067	− 0.25	− 1.37	0.183
Visual short-term memory	0.30	1.67	0.106	0.45	2.65	0.013	0.19	1.00	0.325
Visual long-term memory	0.20	1.10	0.283	0.28	1.57	0.127	0.35	1.96	0.061
Visuospatial perception	− 0.32	− 1.81	0.082	− 0.53	− 3.27	0.003	− 0.43	− 2.53	0.017
Visuomotor processing speed	0.26	1.46	0.155	0.29	1.61	0.119	0.20	1.11	0.278
Manual dexterity	0.06	0.30	0.766	0.27	1.51	0.142	0.19	1.04	0.308
**Syntactic processing**									
Syntactic processing	− 0.54	− 3.43	0.002	− 0.47	− 2.80	0.009	− 0.50	− 3.07	0.005

Note: *n* = 30.

*β* standardized regression coefficient, *p-value* significance in *t* test.

**Figure 1:**
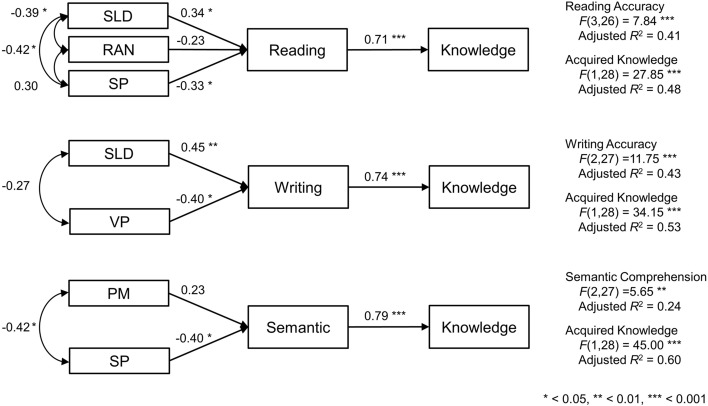
Illustration of the dimension-specific models of Japanese kanji acquisition: the results of regressions in path analyses that include each dimension of kanji abilities, their cognitive underpinnings, and acquired knowledge. Numbers on straight arrows indicate standardized regression coefficients, whereas those on curved double-headed arrows indicate correlation coefficients. The results of *F* tests and adjusted *R*^2^ are shown on the right of the figures. *SLD* sound–letter decoding, *RAN* rapid automatized naming, *SP* syntactic processing, *VP* visuospatial perception, *PM* phonological short-term memory.

Then, we administered step-wise linear regression analyses that included all significant predictors of each dimension of kanji abilities, which were identified in simple linear regressions. The final model demonstrated that the combination of sound–letter decoding (*β* = 0.34, *t*(26) = 2.02, *p* = 0.054), rapid automatized naming (*β* = − 0.23, *t*(26) = − 1.44, *p* < 0.163), and syntactic processing (*β* = − 0.33, *t*(26) = − 2.06, *p* = 0.049) significantly predicted the scores on reading accuracy of kanji abilities, which accounted for 41% of the variance in this dimension (*F*(3,26) = 7.84, *p* < 0.001, adjusted *R*^2^ = 0.41; see Fig. 1). When letter–sound decoding (*β* = 0.02, *t*(25) = 0.11, *p* < 0.915) was added to this model as a predictor, the variance accounted for was reduced to 39% (*F*(4,25) = 5.66, *p* = 0.002, adjusted *R*^2^ = 0.39). Regarding writing accuracy, the combination of sound–letter decoding (*β* = 0.45, *t*(27) = 3.09, *p* = 0.005) and visuospatial perception (*β* = − 0.40, *t*(27) = − 2.71, *p* = 0.012) was found to account for 43% in this dimension (*F*(2,27) = 11.75, *p* < 0.001, adjusted *R*^2^ = 0.43). When phonological awareness (*β* = 0.10, *t*(26) = 0.64, *p* < 0.526), visual short-term memory (*β* = 0.09, *t*(26) = 0.47, *p* < 0.644), or syntactic processing (*β* = 0.03, *t*(26) = 0.15, *p* < 0.884) was added to this model as a predictor, the variance accounted for was reduced to 41% or less (*F*(3,26) = 7.80, *p* < 0.001, adjusted *R*^2^ = 0.41; *F*(3,26) = 7.68, *p* < 0.001, adjusted *R*^2^ = 0.41; *F*(3,26) = 7.55, *p* < 0.001, adjusted *R*^2^ = 0.40). Regarding semantic comprehension, step-wise linear regression analyses demonstrated that syntactic processing (*β* = − 0.50, *t*(28) = − 3.07, *p* = 0.005) accounted for 22% in this dimension (*F*(1,28) = 9.39, *p* = 0.005, adjusted *R*^2^ = 0.22). However, the combination of phonological short-term memory (*β* = 0.23, *t*(27) = 1.30, *p* = 0.206) and syntactic processing (*β* = − 0.40, *t*(27) = − 2.24, *p* = 0.034) accounted for 24% of the variance of semantic dimension of kanji abilities (*F*(2,27) = 5.65, *p* = 0.009, adjusted *R*^2^ = 0.24).

### Relationships between kanji acquisition and higher-level language skills

The results of path analyses that include each dimension of kanji abilities, and their cognitive predictors, and acquired knowledge or idea density are shown in Table 4. All goodness-of-fit indices demonstrated better fits of the models that assumed predictions from each of three dimensions of kanji abilities to acquired knowledge than the models that assumed correlations between them (see Fig. 1). The χ^2^ statistics and RMSEA estimates were both significantly low for the predictive models (all *p* > 0.05; all *p* close > 0.05) but not for the correlative ones (all *p* ≤ 0.006; all *p* close ≤ 0.009). The AIC values for the former were lower than those for the latter, and the CFI, TLI, and SRMR values also indicated good fits only for the former. In the prediction models, each dimension of kanji abilities predicted acquired knowledge (reading accuracy: *β* = 0.71, *t*(28) = 5.28, *p* < 0.001; writing accuracy: *β* = 0.74, *t*(28) = 5.84, *p* < 0.001; semantic comprehension: *β* = 0.79, *t*(28) = 6.71, *p* < 0.001), and accounted for 48% or larger portion of the variances in that score (reading accuracy: *F*(1,28) = 7.84, *p* < 0.001, adjusted *R*^2^ = 0.48; writing accuracy: *F*(1,28) = 34.15, *p* < 0.001, adjusted *R*^2^ = 0.53; semantic comprehension: *F*(1,28) = 45.00, *p* < 0.001, adjusted *R*^2^ = 0.60). Regarding the correlations among the cognitive predictors, in the model of reading accuracy, better performance on sound–letter decoding was associated with those on rapid automatized naming (*r* = − 0.39, *p* = 0.04) and syntactic processing (*r* = − 0.42, *p* = 0.03), whereas the significant correlation between the latter two was not shown (*r* = 0.30, *p* = 0.11). In the model of semantic comprehension, better performance on syntactic processing was associated with that on phonological short-term memory (*r* = − 0.42, *p* = 0.03). In the model of writing accuracy, the significant correlation between sound–letter decoding and visuospatial perception was not found (*r* = − 0.27, *p* = 0.14). Additionally, mediation analyses by a bootstrapping method showed significant indirect effects of all cognitive predictors on acquired knowledge via each dimension of kanji abilities (sound–letter decoding via reading: *β* = 0.41, *z* = 3.43, *p* = 0.001; rapid automatized naming via reading: *β* = − 0.34, *z* = − 2.68, *p* = 0.007; syntactic processing via reading: *β* = − 0.31, *z* = − 2.54, *p* = 0.012; sound–letter decoding via writing: *β* = 0.44, *z* = 2.60, *p* = 0.009; visuospatial perception via writing: *β* = − 0.36, *z* = − 2.31, *p* = 0.021; phonological short-term memory via semantic comprehension: *β* = 0.29, *z* = 2.37, *p* = 0.018; syntactic processing via writing: *β* = − 0.34, *z* = − 2.28, *p* = 0.023).

**Table 4:** Results of path analyses that test models including kanji abilities, their cognitive underpinnings, and higher-level language skills.

Models	χ^2^	*df*	*p*-value	RMSEA	[90%CI]	*p* close	CFI	TLI	SRMR	AIC
**Reading accuracy (predictors: sound–letter decoding, rapid automatized naming, & syntactic processing)**										
Regression for AK	2.87	3	0.413	0.00	[0.00, 0.30]	0.446	1.00	1.01	0.04	392.17
Correlation with AK	12.42	3	0.006	0.32	[0.15, 0.52]	0.009	0.79	0.31	0.23	401.72
Regression for ID	15.34	3	0.002	0.37	[0.20, 0.56]	0.002	0.70	− 0.02	0.13	157.46
**Writing accuracy (predictors: sound–letter decoding & visuospatial perception)**										
Regression for AK	0.86	2	0.652	0.00	[0.00, 0.28]	0.672	1.00	1.09	0.03	307.26
Correlation with AK	10.40	2	0.006	0.37	[0.17, 0.61]	0.008	0.79	0.37	0.24	316.80
Regression for ID	9.60	2	0.008	0.36	[0.15, 0.60]	0.011	0.73	0.19	0.13	328.09
**Semantic comprehension (predictors: phonological memory & syntactic processing)**										
Regression for AK	4.04	2	0.133	0.18	[0.00, 0.45]	0.153	0.95	0.86	0.07	307.04
Correlation with AK	13.98	2	0.001	0.45	[0.25, 0.68]	0.001	0.72	0.17	0.29	316.98
Regression for ID	13.67	2	0.001	0.44	[0.24, 0.68]	0.002	0.60	− 0.21	0.16	331.12

Note: *n* = 30.

*χ*^*2*^ chi-square statistic, *df* degree of freedom of χ^2^ distribution, *p-value* significance in χ^2^ test, *RMSEA* root mean square error of approximation, *90%CI* 90% confidence interval of RMSEA, *p* close = *p*-value for the test of the close-fit hypothesis that RMSEA ≤ 0.05, *CFI* comparative fit index, *TLI* Tucker–Lewis index, *SRMR* standardized root mean square residual, *AIC* Akaike’s information criterion, *AK* acquired knowledge, *ID* idea density.

However, the predictive models that included each dimension of kanji abilities, their cognitive predictors, and idea density were not statistically supported (see Fig. 1). The *χ*^2^ statistics (all *p* ≤ 0.008) and RMSEA estimates (all *p* close ≤ 0.011), as well as other indices, did not show good fits for these models.

### Indirect effects of kanji abilities on idea density via acquired knowledge

**Figure 2:**
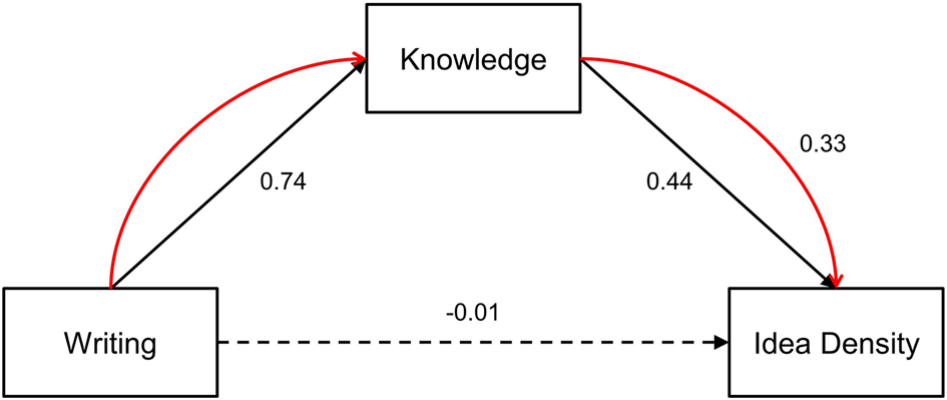
Illustration of the mediation model that includes writing accuracy of kanji abilities (predictor), knowledge acquisition (mediator), and idea density (outcome): Numbers on arrows indicate standardized regression coefficients. Black arrows represent the direct effects. Red arrows represent the indirect effects.

Finally, we administered linear regression analyses with a bootstrapping method to examine the validity of the mediation models that included each dimension of kanji abilities as a predictor, acquired knowledge as a mediator, and idea density as an outcome (see Fig. 2). The results of these analyses are shown in Table 5. Reading accuracy of kanji abilities did not predict idea density directly (*β* = 0.33, *t*(28) = 1.85, *p* = 0.075) or indirectly via acquired knowledge (*β* = 0.28, *z* = 1.57, *p* = 0.116). Writing Accuracy also did not predict idea density directly (*β* = 0.31, *t*(28) = 1.74, *p* = 0.093) but did do so indirectly (*β* = 0.33, *z* = 2.18, *p* = 0.029; see Fig. 2). However, semantic comprehension predicted idea density directly (*β* = 0.38, *t*(28) = 2.17, *p* = 0.039) but not indirectly (*β* = 0.27, *z* = 1.31, *p* = 0.189). When controlling for the effects of acquired knowledge, the direct effects of any dimension of kanji abilities, as well as acquired knowledge, were not significant (all *p* ≥ 0.10). Although acquired knowledge accounted for 16% of the variance in idea density by itself (*F*(1,28) = 6.34, *p* = 0.018, adjusted *R*^2^ = 0.16; *β* = 0.43, *t*(28) = 2.52, *p* = 0.018), whenever each dimension of kanji abilities was added to this model as a predictor, the variances accounted for were slightly reduced to 13% or less (reading accuracy: *F*(2,27) = 3.09, *p* = 0.062, adjusted *R*^2^ = 0.13; writing Accuracy: *F*(2,27) = 3.06, *p* = 0.063, adjusted *R*^2^ = 0.12; semantic comprehension: *F*(2,27) = 3.15, *p* = 0.059, adjusted *R*^2^ = 0.13).

**Table 5:** Testing for indirect effects of kanji abilities on idea density via acquired knowledge.

	*β*	Statistic	*p*-value	Adjusted *R*^2^
**Model: ID on acquired knowledge**		*F*(1,28) = 6.34	0.018	0.16
Acquired knowledge	0.43	*t*(28) = 2.52	0.018	
**Model: ID on reading accuracy**		*F*(1,28) = 3.41	0.075	0.08
Reading accuracy	0.33	*t*(28) = 1.85	0.075	
**Model: ID on AK and RA**		*F*(2,27) = 3.09	0.062	0.13
Acquired knowledge	0.39	*t*(27) = 1.60	0.121	
Reading accuracy	0.05	*t*(27) = 0.21	0.834	
Indirect effect of RA through AK	0.28	*z* = 1.57	0.116	
**Model: ID on writing accuracy**		*F*(1,28) = 3.03	0.093	0.07
Writing accuracy	0.31	*t*(28) = 1.74	0.093	
**Model: ID on AK and WA**		*F*(2,27) = 3.06	0.063	0.12
Acquired knowledge	0.44	*t*(27) = 1.70	0.101	
Writing accuracy	− 0.01	*t*(27) = − 0.05	0.960	
Indirect effect of WA through AK	0.33	*z* = 2.18	0.029	
**Model: ID on semantic comprehension**		*F*(1,28) = 4.71	.039	0.11
Semantic comprehension	0.38	*t*(28) = 2.17	0.039	
**Model: ID on AK and SC**		*F*(2,27) = 3.15	0.059	0.13
Acquired knowledge	0.34	*t*(27) = 1.23	0.230	
Semantic comprehension	0.11	*t*(27) = 0.39	0.699	
Indirect effect of SC through AK	0.27	*z* = 1.31	0.189	

Note: *n* = 30.

*β* standardized regression coefficient, *p-value* significance in *t* or *F* test, *Adjusted R*^*2*^ variance explained by predictor(s), *ID* idea density, *AK* acquired knowledge, *RA* reading accuracy, *WA* writing accuracy, *SC* semantic comprehension.

## Discussion

The present study tested hypotheses regarding the predictive relationships among kanji abilities, their cognitive underpinnings, and higher-level language skills in Japanese university students. Our results support all five hypotheses that (1) combinations of multiple cognitive skills predict each dimension of Japanese kanji abilities, i.e., reading accuracy, writing accuracy, and semantic comprehension; (2) whereas phonological processing is a common factor, the other three domains predicted kanji acquisition in a dimension-specific manner; (3) kanji abilities predict acquired knowledge; (4) each dimension of kanji abilities mediates the predictive relationship between its cognitive predictors and acquired knowledge; (5) the writing dimension of kanji abilities predicts idea density indirectly via acquired knowledge. To the best of our knowledge, these findings represent the first reported evidence of dimension-specific relationships of the three dimensions of Japanese kanji abilities with their cognitive predictors, as well as with higher-level language skills.

### Cognitive underpinnings of Japanese kanji abilities

A series of linear regressions identified the multifactorial models of reading, writing, and semantic dimensions of Japanese kanji acquisition (see Fig. 1). Our results showed that the combinations of common and dimension-specific factors account for 24 to 43% of the variances in each dimension of Japanese kanji acquisition, which were equivalent to those reported for children^6,10^. These results support the methodological validity of investigating the cognitive predictors of Japanese kanji abilities in university students.

Phonological processing measures, including sound–letter decoding and phonological short-term memory, were found to predict all the dimensions of kanji abilities. The contribution of this universal factor of literacy acquisition^8,9,63^ has been reported to be relatively small for Japanese kanji reading/writing^6,10,22^, as well as for Chinese^15^. Our results are consistent with those previous findings. Nonetheless, we add a new finding that phonological processing plays a substantial role in the mastery of kanji, with its impact on all of the three dimensions of kanji abilities.

As dimension-specific factors, the following functions were identified: naming speed for reading accuracy, visuospatial processing for writing accuracy, and syntactic processing for reading and semantic comprehension. First, the contribution of naming speed to reading acquisition, which is smaller than that of phonological processing, has been reported across languages^8,9,64^, including Japanese^10^. Therefore, it is recommended that testing naming speed on kana letters and non-words, as well as kana words and sentences, should be employed in the diagnostic process for reading disorders in Japanese children^46^. Consistent with these findings, the significant effects of naming speed measures were found only on the reading dimension in this study. Second, regarding visuospatial processing, visual memory has been reported as a predictor of spelling or writing acquisition in alphabetic orthographies^19,65^, as well as in Japanese^22^. Although this study employed measures of visual short- and long-term memory, referring to those previous findings, their predictive power was slightly smaller than that of visuospatial perception measured by using a simple block design task. These results suggest the language-specificity of this dimension-specific factor underpinning the ability to write visually complex kanji characters accurately. Third, regarding syntactic processing, our results provided the first evidence of a significant relationship with Japanese literacy. Before now, the studies on alphabetic and Chinese orthographies reported that syntactic processing contributed to reading comprehension on the sentence or text level^28,29^. The present study expands these findings to reading accuracy and semantic comprehension on the word level and to Japanese orthography. In addition, these dimension-specific predictors seem to be evidence supporting the multidimensional nature of Japanese kanji abilities.

The multifactorial models identified in this study suggest the possibility that atypical characteristics in phonological processing cause general difficulties in kanji acquisition, whereas atypicalities in naming speed, visuospatial processing, or syntactic processing cause difficulties in specific dimensions of kanji abilities. However, on the other side of the coin, these models suggest that each of the multiple factors could work as a protective factor to compensate for the difficulties in Japanese literacy caused by other factors. Our results imply the necessity of therapeutic or educational strategies according to the assessment of the dimensions and causes of difficulties in Japanese kanji acquisition.

### Effects of kanji acquisition on higher-level language skills

Path analyses showed the contributions of all the dimensions of kanji acquisition to knowledge acquisition. These results are consistent with both our intuitive expectations and the empirical evidence^30–34^. In addition, mediation analyses showed the indirect effects of all the cognitive predictors on acquired knowledge via kanji abilities, suggesting that, whatever their cause, literacy achievement/problems can affect knowledge acquisition during the school years.

A direct contribution of any dimension of kanji acquisition to idea density was not established in the present study. The direct effect of semantic comprehension on idea density was significant but attenuated to a non-significant level when controlling for acquired knowledge. However, as for the contributions of kanji abilities to idea density via acquired knowledge, a significant indirect effect was demonstrated for the dimension of writing (see Fig. 2). These results suggest that handwriting skills on the word level, rather than reading or semantic skills acquired during school years, can promote the development of writing abilities on the text level. We measured idea density from essays that were typed by participants, but not from handwritten ones. Therefore, the impact of handwriting acquisition does not seem to be confined to the process of handwriting per se but to be generalized to higher-level language skills. This finding may also be true for people using other orthographies. Alternatively, this may only be applicable to Japanese people because there are differences between Japanese and other languages, not only in the writing system but also with respect to many other facets^66^. The effects of the three dimensions of literacy acquisition, i.e., reading accuracy, writing accuracy, and semantic comprehension, on the development of higher-level language skills in non-Japanese people should be examined in the future.

In the present day, digital writing devices are increasingly replacing handwriting across the world. In Japan, the frequency of handwriting has decreased^39^, resulting in a dimension-specific reduction of kanji writing skills in adults^27^. Considering the possible impact on not only handwriting acquisition but also the development of language skills, we must carefully discuss the controversial issue of whether these technologies should be applied to early literacy education^67,68^. The ease of typing or other supportive capabilities of digital tools is considered to be advantageous for literacy learning, particularly in children with underdeveloped motor skills^69^ or reading/writing difficulties^70^, e.g., difficulties related to visuospatial processing. However, there is growing evidence demonstrating that the coupling of motor action and perception during handwriting can facilitate literacy acquisition^71–73^, perhaps especially in cases of superior visuospatial processing. The relationship between the acquisition of handwriting on the word level, not reading and semantic comprehension, and higher-level language skills on the text level shown in this study represents evidence supporting the latter view.

Finally, it is also worth mentioning again that idea density, which is employed in this study as a measure of higher-level language skills, has attracted wide attention in the context of its possible function as a protective factor against Alzheimer’s disease^36–38^. The Nun study demonstrated that young adults with higher idea density tended to preserve cognitive function in later life even when Alzheimer’s disease affected their brains^36,37^. This finding could be explained by the “cognitive reserve” hypothesis. This means that attainment of higher language proficiency in early life may increase the brain’s capacity to make efficient use of available cognitive/neural resources and protect against cognitive decline and dementia in later life^74–76^. Incorporating these findings into the interpretation of our current study, we may describe a lifelong trajectory of the effect of literacy acquisition, from basic cognitive skills through basic literacy (especially writing accuracy) acquired during school years to higher-level language skills (culminating in idea density) throughout adulthood and to the “cognitive reserve” as a protective factor against cognitive decline in late life. This theoretical framework implies that basic literacy acquisition may be a good predictor of later cognitive decline/preservation. Before now, most studies of English-speaking elders have focused on reading accuracy in late life and reported that higher performance in this area predicts cognitive preservation^77,78^. Our current results, which showed the impact of basic handwriting skills on higher-level language skills, might suggest that the domain of writing is also worth investigating, going back to the original Nun study which analyzed autobiographies handwritten by nuns^36^.

### Limitations

First, the results of this cross-sectional study do not exactly examine longitudinal predictions. To clarify the predictive relationships among basic cognitive skills assessed in early childhood, literacy and knowledge acquisition during school years, and idea density in young adults, longitudinal investigations following participants over about one and a half decades are needed. Second, the participants in this study were university students with normal or higher literacy achievement. To generalize the interpretation of our results to those with linguistic difficulties, investigations of a population with a wider range of linguistic abilities are required. Third, the relatively small sample size allowed us to administer statistical analyses that included only one dimension of kanji abilities and was not large enough to support modeling to include all dimensions. Large-sample investigations into more complex relationships among the variables focused on in this study are expected in the future. Fourth, the ceiling effects of a few cognitive measures possibly influenced the results of the statistical analyses. The high accuracy (mean = 97.23%; half of the participants achieved a perfect score) of the measure of phonological awareness, which is seen as the key predictor of literacy acquisition in alphabetic orthographies, suggests the possibility of underestimating the effect of this skill. For examining the effects of phonological awareness on higher-level language skills, e.g., text reading/writing, the development of measures that can assess individual differences in this skill in adults is required. Finally, this study did not measure basic writing skills with digital devices. In the future, it is expected that tests, especially standardized tests like the KABC-II^43^, to measure basic digital writing acquisition will be developed to clarify the relationship between digital writing skills and basic literacy, including handwriting, or between digital writing skills and higher-level language skills.

## Conclusion

The current study revealed the relationships of Japanese literacy with its cognitive underpinnings as well as with higher-level language skills based on the three-dimensional view of kanji abilities, i.e., reading accuracy, writing accuracy, and semantic comprehension. First, among basic cognitive skills, naming speed, visuospatial processing, and syntactic processing underpin kanji abilities in a dimension-specific manner, whereas phonological processing plays the role of a common factor. Second, although all the dimensions of kanji abilities predicted acquired knowledge equally, higher-level writing skills on the text level measured as idea density were predicted by only the writing dimension indirectly via acquired knowledge. These results imply that writing acquisition during school years has a key role in the development of language skills through to adulthood. These findings warrant further research on the effect of handwriting practices, such as those investigating the younger population with linguistic difficulties, as well as those investigating the potential of the cognitive reserve to protect against the development of dementia in later life.

## Data Availability

The data analyzed in this study are available from the corresponding author upon reasonable request.

## References

[1] Nag S, Caravolas M, Snowling MJ (2011). Beyond alphabetic processes: Literacy and its acquisition in the alphasyllabic languages. Read. Writ..

[2] Hanley JR, Snowling MJ, Hulme C (2005). Learning to read in Chinese. The Science of Reading: A Handbook.

[3] Ministry of Education, Culture, Sports, Science and Technology. *Elementary School Curriculum Guidelines* (*Syogakko-gakusyu-shido-yoryo*; in Japanese). http://www.mext.go.jp/a_menu/shotou/new-cs/youryou/syo/index (2008).

[4] Ministry of Education, Culture, Sports, Science and Technology. *High school Curriculum Guidelines* (*Kotogakko-gakusyu-shido-yoryo*; in Japanese). http://www.mext.go.jp/a_menu/shotou/new-cs/youryou/1304427 (2009).

[5] Amano K (1988). Phonemic analysis and literacy acquisition in children (*Onin-Bunsheki-to-Kodomo-no-literacy-no-Shutoku*; in Japanese). Kyoiku Shinrigaku Nenpo.

[6] Inoue T, Geurgiou GK, Muroya N, Maekawa H, Parrila R (2017). Cognitive predictors of literacy acquisition in syllabic hiragana and morphographic kanji. Read. Writ..

[7] Kobayashi MS, Haynes CW, Macaruso P, Hook PE, Kato J (2005). Effects of mora deletion, nonword repetition, rapid naming, and visual search performance on beginning reading in Japanese. Ann. Dyslexia.

[8] Peterson RL, Pennington BF (2015). Developmental dyslexia. Annu. Rev. Clin. Psychol..

[9] Peterson RL, Pennington BF (2012). Developmental dyslexia. Lancet.

[10] Uno A, Wydell TN, Haruhara N, Keneko M, Shinya N (2009). Relationship between reading/writing skills and cognitive abilities among Japanese primary-school children: Normal readers versus poor readers (dyslexics). Read. Writ..

[11] Bishop D, Rutter M, Rutter M, Bishop DVM, Pine DS, Scott S, Stevenson J, Taylor E, Thapar A (2008). Neurodevelopmental disorders: Conceptual issues. Rutter’s Child and Adolescent Psychiatry.

[12] Pennington BF (2006). From single to multiple deficit models of developmental disorders. Cognition.

[13] Moll K, Ramus F, Bartling J, Bruder J, Kunze S, Neuhoff N (2014). Cognitive mechanisms underlying reading and spelling development in five European orthographies. Learn. Inst..

[14] Ziegler JC, Bertrand D, Toth D, Csepe V, Reis A, Faísca L (2010). Orthographic depth and its impact on universal predictors of reading: A cross-language investigation. Psychol. Sci..

[15] McBride-Chang C, Cho J-R, Liu H, Wagner RK, Shu H, Zhao A (2005). Changing models across cultures: Associations of phonological awareness and morphological structure awareness with vocabulary and word recognition in second graders from Beijing, Hong Kong, Korea, and the United States. J. Exp. Child Psychol..

[16] Facoetti A, Corradi N, Ruffino M, Gori S, Zorzi M (2010). Visual spatial attention and speech segmentation are both impaired in preschoolers at familial risk for developmental dyslexia. Dyslexia.

[17] Bosse ML, Tainturier MJ, Valdois S (2007). Developmental dyslexia: The visual attention span deficit hypothesis. Cognition.

[18] Franceschini S, Gori S, Ruffino M, Pedroll K, Facoetti A (2012). A causal link between spatial attention and reading acquisition. Curr. Biol..

[19] Niolaki GZ, Masterson J (2012). Transfer effects in spelling from transparent Greek to opaque English in seven-to-ten-year-old children. Biling.

[20] Yang L-Y, Guo J-P, Richman LC, Schmidt FL, Gerken KC, Ding Yi (2013). Visual skills and Chinese reading acquisition: A meta-analysis of correlation evidence. Educ. Psychol. Rev..

[21] Wolf M, Bowers PG (1999). The double-deficit hypothesis for the developmental dyslexias. J. Educ. Psychol..

[22] Koyama MS, Hansen PC, Stein JF (2008). Logographic Kanji versus phonographic Kana in literacy acquisition. Ann. N. Y. Acad. Sci..

[23] Georgiou GK, Torppa M, Manolitsis G, Lyytinen H, Parrila R (2012). Longitudinal predictors of reading and spelling across languages varying in orthographic consistency. Read. Writ..

[24] Iwata M (1984). Kanji versus Kana: Neuropsychological correlates of the Japanese writing system. Trends Neurosci..

[25] Sakurai Y (2004). Varieties of alexia from fusiform, posterior inferior temporal and posterior occipital gyrus lesions. Behav. Neurol..

[26] Sakurai Y, Mimura I, Mannen T (2008). Agraphia for kanji resulting from a left posterior middle temporal gyrus lesion. Behav. Neurol..

[27] Otsuka S, Murai T (2020). The multidimensionality of Japanese kanji abilities. Sci. Rep..

[28] Brimo D, Apel K, Fountain T (2017). Examining the contributions of syntactic awareness and syntactic knowledge to reading comprehension. J. Res. Read..

[29] Yeung P-S, Ho CS-H, Chik PP-M, Lo L-Y, Luan H, Chan DW-O, Chung KK-H (2011). Reading and spelling Chinese among beginning readers: What skills make a difference?. Sci. Stud. Read..

[30] Cain K, Oakhill J (2011). Matthew effects in young readers: Reading comprehension and reading experience aid vocabulary development. J. Learn. Disabil..

[31] Duff D, Tomblin JB, Catts H (2015). The influence of reading on vocabulary growth: A case for a Matthew effect. J. Speech Lang. Hear. Res..

[32] Stanovich KE (1986). Matthew effects in reading: Some consequences of individuals differences in the acquisition of literacy. Read. Res. Quart..

[33] Cordoni BK, O’Donnell JP, Ramaniah NV, Kurtz J, Rosenshein K (1981). Wechsler adult intelligence score patterns for learning disabled young adults. J. Learn. Disabil..

[34] Moura O, Simões MR, Pereira M (2013). WISC-III cognitive profiles in children with developmental dyslexia: Specific cognitive disability and diagnostic utility. Dyslexia.

[35] Farias ST, Chand V, Bonnici L, Baynes K, Harvey D, Mungas D (2012). Idea density measured in late life predicts subsequent cognitive trajectories: Implications for the measurement of cognitive reserve. J. Gerontol. B.

[36] Snowdon DA, Kemper SJ, Mortimer JA, Greiner LH, Wekstein DR, Markesbery WR (1996). Linguistic ability in early life and cognitive function and Alzheimer’s disease in late life: Findings from the Nun Study. JAMA.

[37] Iacono D, Markesbery WR, Gross M, Pletnikova O, Rudow G, Zandi P, Troncoso JC (2009). The Nun study: Clinical silent AD, neuronal hypertrophy, and linguistic skills in early life. Neurology.

[38] Engelman M, Agree EM, Meoni LA, Klag MJ (2010). Propositional density and cognitive function in later life: Findings from the precursors study. J. Gerontol. B. Psychol. Sci. Soc. Sci..

[39] Ministry of Internal Affairs and Communications. *Communications Usage Trend Survey in 2016* (*Heisei-26-nen-tsushin-riyo-dokotyosa-hokokusyo-setai-hen*; in Japanese). http://www.soumu.go.jp/johotsusintokei/statistics/pdf/HR201600_001.pdf (2017).

[40] Agency for Cultural Affairs. *Public Opinion Poll on Japanese Language in 2012 (Heisei-24-nendo-kokugo-ni-kansuru-yoron-chosa; in Japanese)*. http://www.bunka.go.jp/tokei_hakusho_shuppan/tokeichosa/kokugo_yoronchosa/pdf/h24_chosa_kekka.pdf (2013).

[41] Fujita K, Maekawa H, Dairoku H, Yamanaka K (2006). Japanese Version of the Wechsler Adult Intelligence Scale, 3rd edition (WAIS-III).

[42] Wechsler D (1997). Wechsler Adult Intelligence Scale-Third Edition (WAIS-III).

[43] Kaufman AS, Kaufman NL (2004). Kaufman Assessment Battery for Children-Second Edition (KABC-II).

[44] Okumura T, Kawasaki A, Nishioka Y, Wakamiya E, Miura T, Tamai H (2014). Comprehensive Assessment of Reading Domains guidebook.

[45] Wolf M, Denckla M (2005). The Rapid Automatized Naming and Rapid Alternating Stimulus Tests (RAN/RAS).

[46] Inagaki M, Koeda T, Koike H (2010). Practical Guidelines for Diagnosis and Treatment of Specific Developmental Disorders.

[47] Japan Society for Higher Brain Dysfunction (2006). Clinical Assessment for Attention.

[48] Sugishita M (2001). Japanese Version of the Wechsler Memory Scale-Revised (WMS-R).

[49] Wechsler D, Stone CP (1987). Wechsler Memory Scale-Revised (WMS-R).

[50] Meyers JE, Meyers KR (1995). Rey Complex Figure Test and Recognition Trial (RCFT); Professional Manual.

[51] Shah A, Frith U (1993). Why do autistic individuals show superior performance on the block design task?. J. Child Psychol. Psychiatry.

[52] Costa LD, Vaughan HG, Levita E, Farber N (1963). Purdue Pegboard as a predictor of the presence and laterality of cerebral lesions. J. Consult. Psychol..

[53] Bannatyne A (1974). Programs, materials and techniques. J. Learn. Disabil..

[54] De Clercq-Quaegebeur M, Casalis S, Lemaitre M-P, Bourgois B, Getto M, Vallée L (2010). Neuropsychological profile on the WISC-IV of French children with dyslexia. J. Learn. Disabil..

[55] Poletti M (2016). WISC-IV intellectual profiles in Italian children with specific learning disorder and related impairments in reading, written expression, and mathematics. J. Learn. Disabil..

[56] Covington MA (2012). CPIDR 5.1 User Manual.

[57] Shibata D, Ito K, Nagai H, Okahisa T, Kinoshita A, Aramaki E (2018). Idea density in Japanese for the early detection of dementia based on narrative speech. PLoS ONE.

[58] R Core Team. *R: A Language and Environment for Statistical Computing*. R Foundation for Statistical Computing, Vienna, Austria. https://www.R-project.org (2019).

[59] Browne MW, Cudeck R, Bollen KA, Long JS (1993). Alternative ways of assessing model fit. Testing Structural Equation Models.

[60] Brown TA (2006). Confirmatory Factor Analysis for Applied Research.

[61] Hu LT, Bentler PM (1999). Cutoff criteria for fit indexes in covariance structure analysis: Conventional criteria versus new alternatives. Struct. Equ. Modeling.

[62] Klein RB (2011). Principles and Practice of Structural Equation Modeling (third edition).

[63] Perfetti C, Cao F, Booth JR (2013). Specialization and universal in the development of reading skill: How Chinese research informs a universal science of reading. Sci. Stud. Read..

[64] Ho CS-H, Chan DW-O, Lee S-H, Tsang S-M, Luan VH (2004). Cognitive profiling and preliminary subtyping in Chinese developmental dyslexia. Cognition.

[65] Bourke L, Davies SJ, Sumner E, Green C (2014). Individual differences in the development of early writing skills: Testing the unique contribution of visuo-spatial working memory. Read. Writ..

[66] Motokawa T (1989). Sushi science and hamburger science. Perspect. Biol. Med..

[67] Mangen A, Balsvik L (2016). Pen or keyboard in beginning writing instruction? Some perspectives from embodied cognition. Trends Neurosci. Educ..

[68] Wollscheid S, Sjaastad J, Tømte C (2016). The impact of digital devices vs. Pen(cil) and paper on primary school students’ writing skills: A research review. Comput. Educ..

[69] Genlott AA, Grönlund Å (2013). Improving literacy skills through learning reading by writing: The iWTR method presented and tested. Comput. Educ..

[70] Morphy P, Graham S (2012). Word processing programs and weaker writers/readers: A meta-analysis of research findings. Read. Writ.

[71] Longcamp M, Zerbato-Poudou M-T, Veley J-L (2005). The influence of writing practice on letter recognition in preschool children: A comparison between handwriting and typing. Acta Psychol..

[72] Longcamp M (2008). Learning through hand- or typewriting influences visual recognition of new graphic shapes: Behavioral and functional imaging evidence. J. Cogn. Neurosci..

[73] Kiefer M (2015). Handwriting or typewriting? The influence of pen- or keyboard-based writing training on reading and writing performance in preschool children. Adv. Cogn. Psychol..

[74] Stern Y (2002). What is cognitive reserve? Theory and research application of the reserve concept. J. Int. Neuropsychol. Soc..

[75] Whalley LJ, Deary IJ, Appleton CL, Starr JM (2004). Cognitive reserve and the neurobiology of cognitive aging. Ageing Res. Rev..

[76] Milgram NW, Siwak-Tapp CT, Araujo J, Head E (2006). Neuroprotective effects of cognitive enrichment. Ageing Res. Rev..

[77] Manly JJ, Schupf N, Tang MX, Stern Y (2005). Cognitive decline and literacy among ethnically diverse elders. J. Geriatr. Psychiatry Neurol..

[78] Manly JJ, Touradji P, Tang MX, Stern Y (2003). Literacy and memory decline among ethnically diverse elders. J. Clin. Exp. Neuropsychol..

